# Safety and Lack of Negative Effects of Wearable Augmented-Reality Social Communication Aid for Children and Adults with Autism

**DOI:** 10.3390/jcm7080188

**Published:** 2018-07-30

**Authors:** Ned T. Sahin, Neha U. Keshav, Joseph P. Salisbury, Arshya Vahabzadeh

**Affiliations:** 1 Brain Power, LLC, Cambridge, MA 02142, USA; neha@brain-power.com (N.U.K.); joey@brain-power.com (J.P.S.); arshya@brain-power.com (A.V.); 2 Department of Psychology, Harvard University, Cambridge, MA 02138, USA; 3 Psychiatry Academy, Massachusetts General Hospital, Boston, MA 02114, USA

**Keywords:** Autism, autism spectrum disorder, augmented reality, technology, Google Glass, social communication, safety, smartglasses, digital health, Amazon, Amazon Web Services, Google

## Abstract

There is a growing interest in the use of augmented reality (AR) to assist children and adults with autism spectrum disorders (ASD); however, little investigation has been conducted into the safety of AR devices, such as smartglasses. The objective of this report was to assess the safety and potential negative effects of the *Empowered Brain system*, a novel AR smartglasses-based social communication aid for people with ASD. The version of the Empowered Brain in this report utilized Google Glass (Google, Mountain View, CA, USA) as its hardware platform. A sequential series of 18 children and adults, aged 4.4 to 21.5 years (mean 12.2 years), with clinically diagnosed ASD of varying severity used the system. Users and caregivers were interviewed about the perceived negative effects and design concerns. Most users were able to wear and use the Empowered Brain (*n* = 16/18, 89%), with most of them reporting no negative effects (*n* = 14/16, 87.5%). Caregivers observed no negative effects in users (*n* = 16/16, 100%). Most users (77.8%) and caregivers (88.9%) had no design concerns. This report found no major negative effects in using an AR smartglasses-based social communication aid across a wide age and severity range of people with ASD. Further research is needed to explore longer-term effects of using AR smartglasses in this population.

## 1. Introduction

Autism Spectrum Disorder (ASD) is a neurodevelopmental disorder affecting 1 in 68 children in the United States [1] and is characterized by social communication impairment as well as the presence of a restricted and/or repetitive range of interests and behaviors [2]. The rising prevalence of ASD has increased the demand for educational and behavioral services, often exhausting these limited resources [3,4]. There has been considerable interest in the development and study of technology-aided approaches for the social, cognitive, and behavioral challenges related to ASD [5,6,7]. Technology-aided approaches may be especially suitable for people with ASD given that some of these individuals may show a natural propensity to utilize digital tools [8], display a fondness for electronic media [9], express a preference for standardized and predictable interactions [8], enjoy game-like elements [10], and/or favor computer-generated speech [11]. However, technology may also have negative effects in some people with ASD. Individuals may develop problematic video game use [12], and can become agitated or disruptive when attempting to disengage from video games [12]. Anecdotally, many caregivers describe meltdowns and other episodes of behavioral dysregulation in children with ASD when attempting to stop them playing on smartphone and/or tablets [13].

Evidence suggests that a broad range of technology-aided interventions, such as those using computer programs and virtual reality (VR), may be effective for people with ASD [5]. Technology-based interventions have been found to be beneficial for improving a wide range of skills and behaviors, including aiding social and emotional skills [14,15], communication ability [15], academics [16], employment proficiencies [6], and challenging behaviors [14]. Additionally, teaching of socio-emotional skills to children and adolescents with ASD is important, as it can help them prepare for the workplace [6,17]. This is a key consideration, as the current rates of unemployment and underemployment among people with ASD are high [18], and the social demand of job and job interviews have been identified as a key challenge [19].

**Figure 1:**
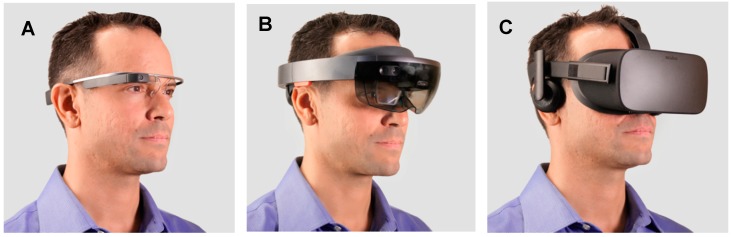
Head-worn Computers or Displays Vary in Size, Weight, and Face-Obstruction. (**A**) Glass Explorer Edition (originally known as Google Glass): AR smartglasses with a fully stand-alone onboard computer (weight 42 grams). (**B**) Microsoft Hololens: AR headset with a fully stand-alone onboard computer and depth camera (weight 579 grams). (**C**) Oculus Rift: VR headset display, which must be tethered continuously to a powerful computer to drive it (weight 470 grams). VR headsets and some AR devices are large, heavy, and block the social world considerably. Image depicts the study author, NTS.

There is particular interest in interventions that help users learn while continuing to interact with the people and environment around them. Learning socio-emotional skills in real life settings (such as in social skills groups) may increase the chance that these behaviors will generalize to the challenges of daily life [20]. Augmented reality (AR) is a technology that holds considerable promise in this regard, allowing users to see and interact with the real world around them, while virtual objects and audio guidance are provided through a visual overlay and audio speakers (Figure 1A,B). In contrast, current VR headsets place users and their senses into an entirely virtual world, while simultaneously removing their ability to see and sense real-world stimuli, hazards, and social situations around them (Figure 1C). In contrast to VR headsets, AR allows users to see their real-world environment, allowing them to navigate an environmental hazard more readily, or to socially engage with another person. Nonetheless, AR incorporates many of the features of VR that are thought to make VR technology well suited to the creation of learning tools for people with ASD [21], including being a primarily visual and auditory experience, being able to individualize the experience, promoting generalization and decreasing rigidity through subtle, gradual modifications of the experience [21].

AR experiences can also be easily modified and personalized for each individual, an important consideration given that many people with ASD exhibit intense interest in a restricted range of topics and may experience extreme distress if changes to their routine/environment occur [2]. AR experiences are also not restricted solely to real-world limitations on time, space, and resources. For instance, users may have the opportunity to interact with objects or experiences from historical or fantasy worlds, or a simplified and cartoon-like interaction, where the sensory and perceptual experiences may be reduced in complexity and/or magnitude.

Most ASD-related research into AR has focused on the use of smartphone- and/or tablet-based apps. While research has been limited, AR apps on smartphones/tablets have been shown to improve selective and sustained attention [22], attention to social cues [23], the ability to understand emotions and facial expressions in storybook characters [23], and navigating the physical world when attempting to find employment opportunities [24]. However, smartphone-based AR may carry with it a risk of negative effects, including grip and postural strain, minor falls, and falls leading to major trauma and blindness [25,26].

While AR has been investigated as an educational medium for ASD children for at least a decade [27], minimal research has been conducted into the safety of head-mounted AR in ASD populations. This has potential implications, as head-mounted AR, in particular, smartglasses, may offer advantages compared to smartphone- or tablet-based AR and may be the optimal future platform for AR [28,29]. The generalized use of AR smartglasses may still be in its infancy, but the use of such devices will be fueled by their ability to improve social interactions and relationships, making life more efficient, and provide enjoyment and fun to the user [30]. AR smartglasses may also be beneficial tools for clinical research. AR smartglasses contain a wide array of sensors. These are intended to allow for basic features, such as gesture-based control of the devices (to make up for the lack of keyboards and traditional input devices). However, we have shown that these sensors can also be used creatively to collect quantitative data that may help assess brain function [31]. Analysis of quantitative data from sensors in smart-devices may help to advance digital phenotyping of neurobehavioral conditions [31]. To our knowledge, we have published the first reports on the use of AR smartglasses in children with ASD [31,32,33,34,35,36].

Even in VR, about which there are many more reports in the literature, there are very few reports on people with ASD using modern VR headsets [37,38]. Therefore, it would be useful to understand how children and adults with ASD respond to AR smartglasses, particularly when the smartglasses function as an assistive device loaded with specialized assistive social and behavioral coaching software [31]. Of primary importance in assessing a new assistive technology is the assessment of (a) the safety of such an approach and (b) any potential negative effects.

There are both human and device factors that make it conceivable that even commercially-available AR technology could elicit concerns regarding safety or negative effects when applied as an assistive technology for this special population.

Firstly, in regard to human factors, it has been widely reported that people with ASD have a range of sensory [2,39], motor [40], and cognitive challenges [41,42], as well as strong negative reactions to transitions [43]. More specifically, atypical reactivity to sensory inputs, such as touch, sound, temperature, and sight, is a diagnostic criterion of ASD [2], affecting up to 93% of people with the condition [44]. Altered sensory reactivity is also highly heterogeneous in the ASD population. Each member of this diverse spectrum may be affected across several senses with hyper- or hypo-sensitivities, representing a complex matrix of sensory subtypes [39]. It is therefore important to determine whether individuals can safely use smartglasses for an extended period and to monitor how they respond to visual, auditory, and vibrotactile cues, delivered through the device.

Secondly, there may be safety concerns because ASD is often associated with altered motor movements, such as repetitive behaviors (a “core” symptom of ASD) [2] or impairments of motor coordination [40]. It is thus important to assess if such motor challenges may lead to falls or injury when people with ASD utilize AR smartglasses [40].

Thirdly, people with ASD may differ in their ability to remain attentive and focus on using smartglasses as part of social communication training, especially given the high rate of comorbidity between ASD and attention deficit hyperactivity disorder [45]. Some individuals may find themselves becoming distracted when using AR or in the process of becoming familiar with using AR while simultaneously navigating the real world [46].

These attentional difficulties may compound the motor coordination challenges in ASD, as mentioned above, increasing the potential of AR smartglasses use to cause falls and/or trips. Additionally, over 30% of children with ASD demonstrate wandering/elopement behavior, and it would be prudent to investigate any technology that would affect their perception, attention, and ability to avoid hazards [47].

Finally, people with ASD may face major challenges in coping with transitions in activities [2,48] and have demonstrated oppositional defiant behaviors and aggression when asked to stop playing video games [12] or stop using a piece of technology [13]. This suggests a possible risk of meltdown when an AR session is ended, though it remains to be seen whether stopping the use of smartglasses results in less difficulty than when stopping the use of a smartphone or tablet (which may be more engrossing or cognitively demanding).

Instruction manuals for AR smartglasses are an additional indication that there may be device-related factors that result in risks. For instance, the Microsoft HoloLens manual identifies the potential side effects as nausea, motion sickness, dizziness, disorientation, headache, fatigue, eye strain, dry eyes, and seizures [49], although their occurrence among users with ASD has not been studied. There is evidence that Google Glass can reduce the visual field of users’ right eye, although this effect seems mostly attributable to the frame of the glasses [50].

Few studies have investigated how these new AR devices may impact the perceptual abilities of regular users, raising concerns that some individuals may become distracted, have altered reaction times, misjudge hazards in the real-world, and/or experience altered distance and speed perception [46].

AR may share a subset of the risks of VR, and VR research has reported potential side effects that include eye strain, headache, and disorientation during the use of a VR headset [51]. However, there have been continuous advances in VR technology, and a recent study noted that people with ASD experienced relatively few negative effects when using a VR headset of the modern generation [38]. 

Assessing negative effects in people with ASD is not a simple undertaking, given that these individuals have challenges in communicating their experiences. It is therefore important to explicitly ask for their feedback, and seek feedback from their caregivers to have a more comprehensive method for detecting any negative effects.

## 2. Aims of Research

Given the potential for AR smartglasses to be used in people with ASD, and yet the uncertainty as to whether this technology would be safe in this population, we studied a specific AR smartglasses technology in 18 children and adults with ASD. The system used in this study was the *Empowered Brain, previously called the Brain Power Autism system (BPAS)* (Brain Power, LLC, Cambridge, MA, USA) [31].

### 2.1. The Empowered Brain System

The Empowered Brain is a social communication aid that consists of AR smartglasses with apps that allow children and adults with ASD to coach themselves on important socio-emotional and cognitive skills [31,32]. The typical session length of a Empowered Brain intervention is 10 min in duration, and a session is typically conducted once or twice a day.

Users of the Empowered Brain learn life skills through gamified interactions and a combination of intrinsic and extrinsic rewards for successfully completing tasks. In certain situations, such as coaching of appropriate face-directed gaze and emotion recognition, the Empowered Brain is designed to be used while the user is interacting with another person. The system was designed using serious game principles and an iterative process, where continuous feedback from people with ASD, clinicians, neuroscientists, educators, caregivers, design specialists, and engineers helped to develop the system that was used in this report. Additionally, the facial affective analytics component of the Empowered Brain was developed in partnership with Affectiva, an emotion artificial intelligence company. Other artificial intelligence functions of the Empowered Brain (deep learning and machine learning) have been developed through a partnership with Amazon (Seattle, WA, USA). The work was also made possible by Google, Inc., (Mountain View, CA, USA), now known as Alphabet, Inc., (Mountain View, CA, USA), who provided substantial hardware as well as guidance in engineering. Engineering guidance, including how best to develop apps that would be accessible to a diverse set of users, was provided, in part, through the Glass Enterprise Partnership Program.

The Empowered Brain is designed to be accessible to people with ASD and to minimize potential negative effects. A number of elements were used to achieve this, including, but not limited to, the use of calming tones and emotional artificial intelligence, the minimization of audio and visual sensory load, graduated transitions between learning segments, and the modification of the functionality of the tactile input surfaces of the smartglasses. In this study, we focused on understanding the safety and potential negative effects that children and adults with ASD may experience as they use AR smartglasses that are delivering cognitive and social self-coaching apps.

### 2.2. Technical Specifications of Empowered Brain

The Empowered Brain in this report is based on Google Glass Explorer Edition (Google Glass XE) (Figure 1A) [52]. Google Glass XE are titanium-framed smartglasses, with a high resolution right-sided monocular optical display. Google Glass EE contains a 5-megapixel camera, and can record video at 720p [52]. Audio is generated through a bone conduction transducer on the right side of the smartglasses. It has a lithium ion battery with a capacity of 670 mAH. Battery life in medical settings has been documented as being between 8.5–10 h, although it may be significantly shortened when running high demand applications [53]. The Empowered Brain combines Google Glass XE with a series of apps that help to coach social and emotional skills (summary in Table 1 and [31]).

### 2.3. Face2Face

**Figure 2:**
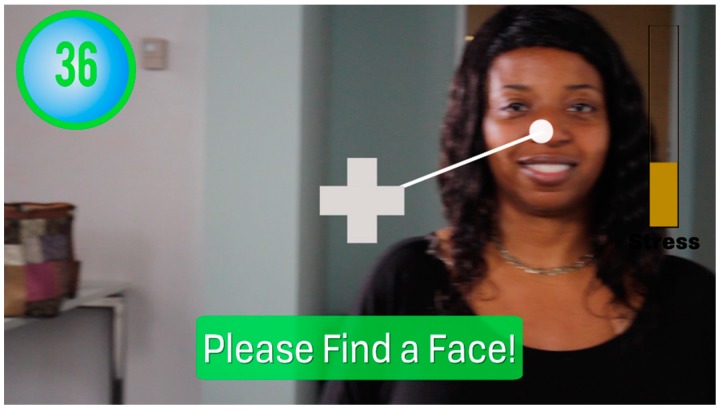
Face2Face module. Representative screen-capture image demonstrating a moment of what a Face2Face user experiences. Face2Face is one of the apps or modules of the Empowered Brain wearable system. This module includes artificial intelligence that finds and tracks faces, and is designed to make an engaging video game-like experience out of learning to direct one’s gaze toward a partner when conversing. Through the computer screen of the wearable smartglasses headset (such as Google Glass), the user gets feedback that encourages face-directed gaze. For instance, in the moment represented here, the user is guided to redirect attention back to the partner’s face via tones, visual words, and a dynamic arrow (drawing on “universal design for learning” by engaging multiple alternative senses and channels of reinforcement simultaneously). When mutual gaze is re-established, the user continues to earn points, stars, and temporary cartoon facemasks for achievement levels.

Human faces are the richest source of socially salient information on humans, information that is crucial to successful social functioning [54]. People with ASD have been found to have a wide range of impairments to their ability to attend to faces, recognize facial emotions, and demonstrate neurotypical patterns of socially-related eye gaze [55,56,57,58,59]. The Empowered Brain includes the Face2Face app, a game-like software that uses a range of strategies to coach users to attend to human faces (Figure 2).

The user, while wearing the Empowered Brain with Face2Face running, sits in front of a human partner who will help to facilitate the interaction. The Face2Face app is able to detect the presence of a human face in its visual field. 

Face2Face determines where the user is looking relative to the partner’s face, and generates a series of AR elements in the user’s field of view (Figure 2). These AR elements, such as guidance arrows and cartoon-like masks, are designed to guide the user to look towards the partner’s face if attention is lost. The guidance arrows help to direct the user towards the partner’s face, dynamically lengthening and shortening in accordance with the user’s head movements. The cartoon-like mask is overlaid over the partner’s face to improve the attention and motivation of the user to move their head in the direction of the face. The cartoon-like mask becomes more translucent as the user moves to look closer to the partner’s face. Based on the user’s performance, points are awarded, and the user can ‘level up’, unlocking further cartoon-like elements. Short auditory tones that correspond with various game events are present throughout the experience, and are delivered through the bone conduction transducer on the right side of the Empowered Brain smartglasses.

### 2.4. Emotion Charades

**Figure 3:**
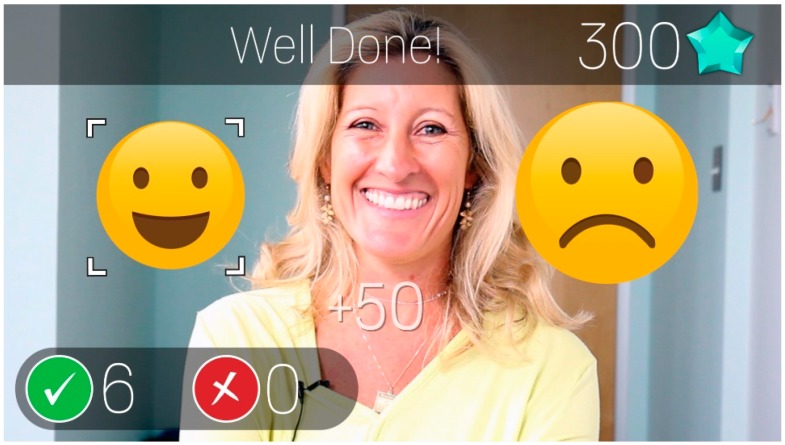
Emotion Charades **module**. Snapshot of what appears on the smartglasses screen during a representative moment during the Emotion Charades module. The moment depicted is immediately after the user has correctly chosen the “happy” emoticon as the one that represents the emotional expression on the face of the partner. The user gets multi-sensory automated feedback, and additionally the partner is cued to give the user specific prompts and mini-exercises to reinforce increasing levels of processing of the target emotions.

The Empowered Brain also includes Emotion Charades, an app that helps to teach human facial emotions through a game-like experience. Emotion Charades, like Face2Face, requires a partner to be present. The focus of the experience is the human-human interaction, with game providing motivation, a semi-scripted paradigm within which to interact, and tracking of progress over time. Emotion Charades, the Empowered Brain can not only detect a human face, but also identify the emotional expression that is being displayed through emotion artificial intelligence technology (Affectiva, Boston, MA, USA). Once an emotion has been detected by the device, two different AR emojis are presented to the user via the private optical display on Glass (Figure 3). One emoji corresponds to the facial expression of emotion that the partner is displaying, while the other does not. The user is asked to identify the correct emoji using a simple left or right head tilt. The head movements are detected by the software using in-built motion sensors of the headset. A correct choice triggers on-screen visual and verbal rewards and an auditory cue. Like Face2Face, short audio cues, corresponding to different game events, are delivered via the bone conduction transducer.

### 2.5. Transition Master

**Figure 4:**
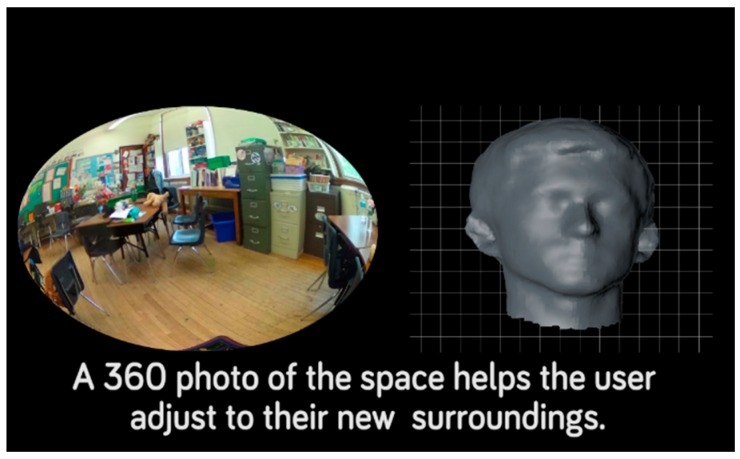
Transition Master Module. Spherical, immersive images can readily be taken of a new place such as a new classroom, or stressful environments such as a crowded or noisy mall or restaurant. These images are displayed by the Empowered Brain headset, offering the user exposure to an unfamiliar setting or context and the ability to practice navigating the environment before visiting it in person.

The Empowered Brain system also incorporates Transition Master, an app that can familiarize a user with a new environment by allowing her or him to interact with and view a 360-degree image of the physical location, as displayed by the optical display of the smartglasses (Figure 4). Transition Master is designed to help users with changes in environment. Many people with ASD commonly experience extreme distress during a change of activity or environment [2]. In the app, the 360-degree image of the “new” location is shown on the optical display. The 360-degree view dynamically changes in real-time with the head movements of the user. Transition Master, unlike Face2Face or Emotion Charades, does not require another person to be a facilitator, or to present a stimulus to the user. Auditory cues and sounds are provided during the experience. The user can tap the headset to transport to other linked rooms or areas when viewing a door, hallway, or other way one would naturally move though the space in reality.

## 3. Methods

The methods and procedures of this study were approved by Asentral, Inc., Institutional Review Board, an affiliate of the Commonwealth of Massachusetts Department of Public Health. The study (2015-405A) was performed in accordance with relevant guidelines and regulations. The study was conducted in accordance with the Declaration of Helsinki.

### User Recruitment

A sequential sample of 18 children and adults with ASD were recruited from a database of individuals who completed a web-based signup form, expressing interest in our study (mean age 12.2 years, range: 4.4–21.5 years; Table 2). Users included males and females, both verbal and non-verbal, and represented a wide range of ASD severity levels. Caregivers confirmed that the participants had received a professional ASD diagnosis.

A Social Communication Questionnaire (SCQ) was completed for all users, with scores ranging from 6 to 28, with an average of 18.8. The SCQ is a validated way to obtain diagnostic and screening information about ASD [60,61]. Information regarding sensory symptoms was available in 14 of the 18 users, with the majority of those users having sensory challenges (*n* = 13/14, 92%).

Written and informed consent was obtained from all adult research participants and from the parents/legal guardians of all minors. Participants between 7 and 17 years-old additionally provided written consent, when appropriate. Every user was accompanied by a caregiver, and participants and caregivers could exit the session at any time and for any reason. Written and informed consent was obtained from all adults and the parents/legal guardians of all minors for the publication of their identifiable images. Consent was obtained for video and audio recording of the sessions. No compensation was offered to any participant or caregiver for taking part in the study, although reimbursement for their parking expenses was offered.

## 4. Exclusions

Individuals who had expressed interest via the website signup, but who had a known history of epilepsy or seizure disorder, were not enrolled in this study. Participants who had an uncontrolled or severe medical or mental health condition that would make participation in the study very difficult were also not enrolled. Two individuals were excluded due to the above criteria.

### Data Collection Procedure

All testing was undertaken in a controlled research environment, and each participant (user) was accompanied to the session by their caregiver. Each user–caregiver dyad was tested separately. A total of 18 user–caregiver dyads participated in the below intervention; 2 were excluded due to meeting the exclusion criteria, stated above.

**Figure 5:**
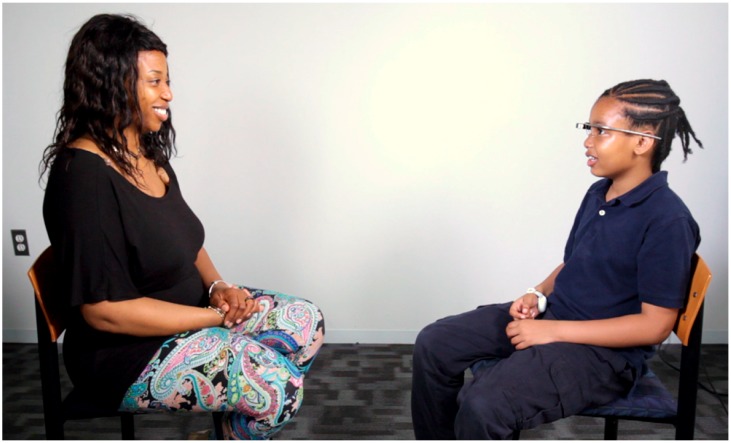
Empowered Brain User-Caregiver Setup. In each session, the participant and caregiver sit facing one another, promoting a ‘heads-up’ social interaction while trialing the apps. Written and informed consent has been obtained for the publication of these images from the depicted adult and from the parents/legal guardians of the minor.

Each user and caregiver was asked to sit on chairs facing one another (Figure 5). A doctoral level clinical researcher oriented users and caregivers to the Empowered Brain hardware [Google Glass XE (Figure 1A)]. Users who could physically wear the smartglasses for at least one minute were allowed to proceed to testing the different Empowered Brain social and cognitive coaching apps (Table 1). The users and caregivers interacted with each other through a series of gamified experiences on the Empowered Brain.

**Figure 6:**
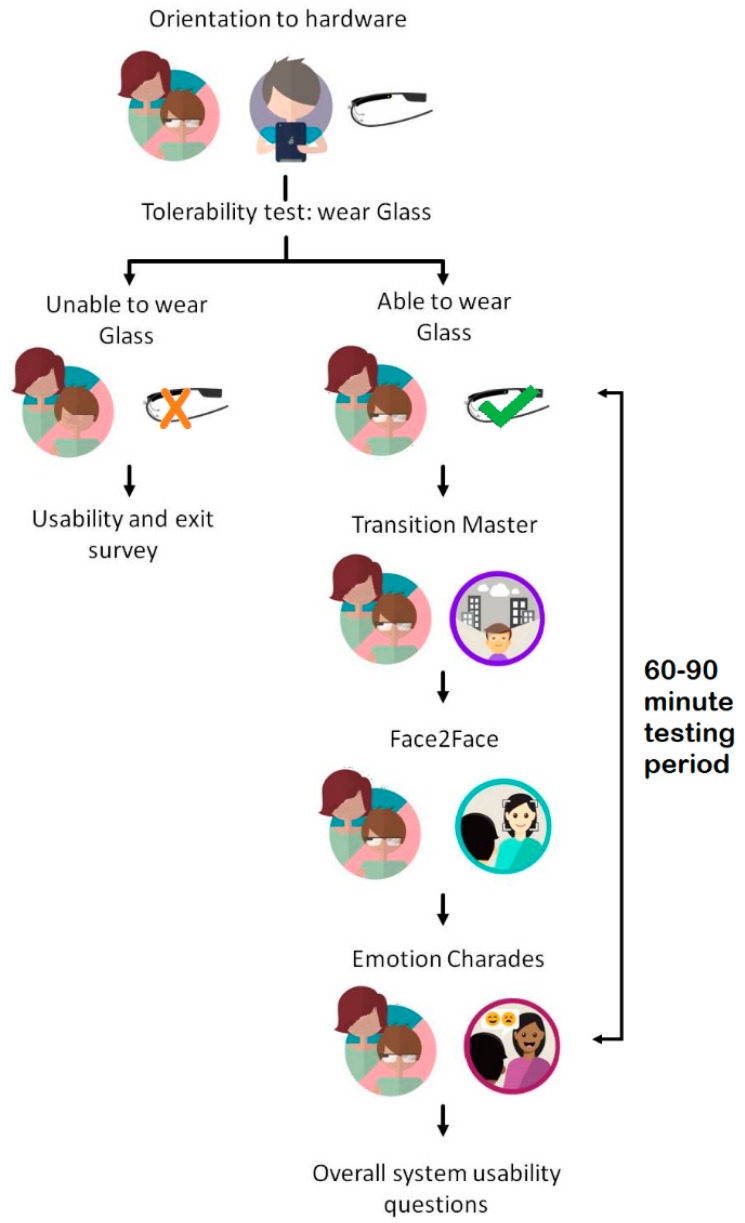
Outline of Phases of Study, including orientation to hardware, initial tolerability test of 1-min duration, followed by 60–90 min multi-module testing period.

**Figure 7:**
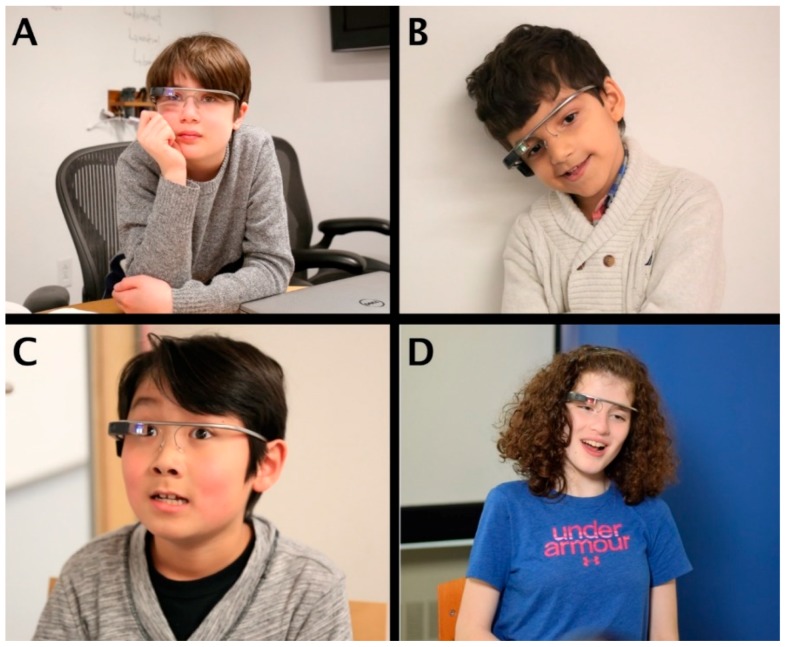
(**A**–**D**) Smartglasses Platform in Use. Four representative trial participants (**A**–**D**) wearing the Empowered Brain. This version of the Empowered Brain used the Glass Explorer Edition device (originally known as Google Glass). Written and informed consent has been obtained from the parents/legal guardians of the minors for the publication of these images.

The experiences were semi-structured in nature, with a total session duration of 60–90 min. The level of variability in the session length, required to use the range of apps, was reflective of the considerable range of ASD severity in the user group. After orientation and an assessment of tolerability, users were able to use Transition Master, then Face2Face, followed by Emotion Charades (Figure 6). Each user experienced each app for approximately 10 min (Figure 7). As previously noted, the Empowered Brain has been designed to be used in 10 min sessions, either once or twice a day. The relatively long duration of testing, relative to real-world use, was chosen in order to more robustly assess the response of users to the technology.

The smartglasses were taken off the user by staff or the caregiver, as required, for the purpose of repositioning, if the Empowered Brain application was to be changed or if there were user/caregiver usability questions.

Following the experience with the system, structured interviews were conducted with users and their caregivers. In the structured interviews, users and caregivers were asked to identify any perceived negative effects of using the system, and could raise concerns or give comments about the design of the smartglasses hardware as well as the apps.

## 5. Results

**Figure 8:**
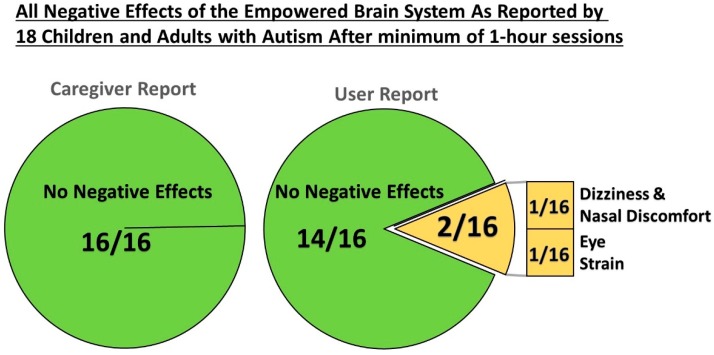
All Reported Negative Effects of the Empowered Brain System. Two users reported a total of three negative effects. One user experienced dizziness and nasal discomfort, and one user experienced eye strain. Caregivers reported that they observed no negative effects on users.

Sixteen of the 18 users (89%) tolerated wearing Empowered Brain smartglasses for at least one minute. The two users who did not tolerate this initial testing did not use Empowered Brain apps. While the two users and their caregivers did not report any adverse effects, the users did not express an interest in wearing the Empowered Brain or continuing the testing session. It was noted that both users were non-verbal, and were relatively young, aged 5.5 and 5.8 years. Of the remaining users, 14 out of 16 users (87.5%), and 16 out of 16 caregivers (100%), reported no minor negative effects, and 100% of caregivers and users reported no major negative effects (Table 3; Figure 8).

Negative effects were determined inductively. The three instances of negative effects were reported by two users. The effects were all mild in nature, transitory in duration, and did not result in session termination. The reported negative effects were one case of dizziness, one case of eye strain, and one instance of initial nasal bridge discomfort. The caregiver of the user experiencing dizziness later explained that the effect may have been related to the user not wearing his/her prescription glasses, and that s/he had previously experienced similar dizziness when s/he had tried a modern VR headset. This same user also experienced initial discomfort with the nose pads, but resolved the discomfort by adjusting the placement of the smartglasses. The user who had complained of eye strain resolved the issue with a 20-s break in testing.

In users who passed the initial tolerability test, the majority of users and their caregivers did not have any design concerns about the system (75% and 87.5% respectively) (Table 3). The only design concern highlighted by users and caregivers was that the smartglasses became warm to the touch during use, although this did not result in any negative effects (Table 4).

## 6. Discussion

The safety and effects of AR smartglasses in children and adults with ASD is an important but poorly researched area, especially given the potential benefits of this technology. People with ASD who use AR smartglasses could potentially experience negative effects due to a range of known device-related factors and ASD-related human factors. ASD-related human factors include challenges in sensory, motor, attentional, and transition-related processes. Device-related factors, as per manufacturer warnings about side effects, include dizziness, headache, and seizures.

This paper explored the use of the Empowered Brain, a novel technological system that uses AR smartglasses to deliver social and cognitive coaching to children and adults with ASD. The focus was on exploring the safety and negative effects of using this technology across a broad age and severity range of children and adults with clinically diagnosed ASD. The duration of use of the Empowered Brain was between 60–90 min, considerably longer than the length of the 10-min intervention that the Empowered Brain has been created to deliver. Additionally, the practicalities of conducting this research involved circumstances that the authors believe could have made the experience more difficult for users than would have been the case had they tested/used the AR smartglasses in a more naturalistic home setting. During the day of testing, users and caregivers were exposed to novel surroundings by attending the research center and being asked to undertake a number of environmental transitions prior to the testing, and users had the additional sensory load of being video and audio recorded while using the Empowered Brain. 

In this context our results are encouraging, and suggest that the majority of people with ASD can use these AR smartglasses without reporting any major negative effects. Of the 16 users who managed to wear and use the Empowered Brain (*n* = 16/18), neither caregivers nor users reported negative effects in 14 cases (*n* = 14/16, 87.5%). In the two individuals who reported negative effects, there were three reported issues: One case of dizziness, one case of eye strain, and one instance of initial nasal bridge discomfort. These negative effects were mild in nature, temporary, and did not lead to the user or caregiver stopping the session. It is important to note that these negative effects were not reported by the caregiver, but rather the participant, further justifying the explicit interviewing of people with ASD in order to understand their experience of this technology. Our participant sample included individuals who had a considerable ASD-related symptom load and those whose symptoms at the time of testing would fall below the typical cut-offs used for ASD-screening via the SCQ. The mean SCQ score of our participants was, however, markedly higher than previously used screening cut-offs. As we previously noted, two individuals were not able to, or did not show interest in, wearing/using the Empowered Brain. This small, but clinically important, group of participants were non-verbal, relatively young and were not reported to have had any negative effects. This may suggest that the technology may be more suited to people with higher-functioning ASD and that some individuals with ASD may struggle to utilize current AR smartglasses, particularly in a research environment.

The participants’ caregivers suggested that an acclimation period to the physical wearing of smartglasses would likely improve the participants’ reaction to the system. 

This study tested the use of this particular technology for a prolonged single intervention session, between 6–9 times the length of the anticipated session length of 10 min. However, the study did not explore the longitudinal use of the technology outside of a controlled research environment. The study was conducted in a controlled research environment, as this allows for a more accurate assessment of participant and caregiver behavior and responses. However, many individuals with ASD receive therapeutic and educational interventions, over a prolonged period of time, in a variety of settings, such as their schools and homes. In this regard, it would be useful to conduct similar research that would incorporate longitudinal assessments in these ecologically valid environments.

The relative lack of negative effects in this AR paradigm is an important finding across such a wide age and severity range of people with ASD, and it indirectly supports recent research demonstrating minimal negative effects when modern VR headsets were used by people with ASD [38]. It was reassuring to see that no major negative effects were reported and, additionally, that no behavioral problems, such as tantrums or meltdowns, occurred when users were asked to stop using the smartglasses during adjustments or application switches, especially given earlier outlined concerns regarding the potential for distress relating to transitions involving technology. Despite the majority of users having sensory sensitivities, there were no concerns regarding sensory-related negative experiences during the use of the Empowered Brain. There were also no falls or motor issues encountered during this study, although our experimental methodology and intervention did not require users to stand, walk, or otherwise demonstrate motor, gait, or balance activities that could be deemed to be a stress test of such activities in this population. Users and caregivers were seated during the experience, and this approach may have intrinsically reduced the risk of falls. AR technologies that require users to attend to the software, while simultaneously requiring users to engage in complex physical and cognitive real-world tasks would need to be more rigorously evaluated. Our prior research has suggested that the use of smartglasses technology alone, without AR, can be used to safely assess balance and complex body movement [1]. Therefore, our findings may not be generalizable to situations where users would be required to engage in notable motor activity in combination with a task-related cognitive load.

There were no design concerns by the majority of caregivers and users. Design concerns were raised by two caregivers and four users, who noticed a feeling of warmth from the external side of the hardware after extended use. However, this did not result in any reported negative effects. User acceptance of design is an important part of any assistive technology experience, so it was useful to know that users and caregivers had few concerns about the design and use of the Empowered Brain.

There remains a critical need to conduct further research to understand the feasibility and safety associated with new emerging technologies, especially those that may be used in vulnerable populations, such as ASD. The use of AR smartglasses may have considerable potential as an augmentative technology in helping people with ASD, particularly when they are shown to be usable and safe in the ASD population and supported by robust evidence of efficacy. While our results suggest that this particular combination of hardware and software is largely devoid of negative effects, our findings may not be generalizable to systems based on other types of AR smartglasses or software apps. Therefore, our findings should not be considered evidence that all AR technologies and software are safe in ASD populations but should rather be considered preliminary evidence that carefully designed technology with user involvement can allow for the safe delivery of specific AR-related interventions.

Additionally, while this report does not identify any short term adverse events, as with any technology, further research is warranted to explore the positive and negative effects of longer term repeated use.

## Figures and Tables

**Table 1:** Intervention focus of Empowered Brain Software Applications.

Empowered Brain App	ASD-Related Challenge	Educational Element	Software Element	Interactivity
**Face2Face**	Reduced attention to faces	Increased attention to human faces	AR guidance of user to the face of facilitator using game-like interface, guidance arrows and cartoon-like masks.	Requires live facilitator to be present. Face of facilitator is utilized by app.
**Emotion Charades**	Difficulty in recognizing facial emotions of others	Improved ability to recognize human facial emotions	App detects human face and identifies emotion displayed. User tilts head corresponding to emotion on human face. Head movement is detected by Empowered Brain (Google Glass) sensors.	Two-person interaction, requires facilitator to be present. Facial emotions of facilitator are utilized by app.
**Transition Master**	Difficulty in handling change of physical environment	Enhanced ability to handle environment/task transitions	App presents user with 360-degree visual image of another environment. User explores environment through head movements that are detected by Empowered Brain sensors.	No interactive facilitator required. User can interact with the environment alone.

**Table 2:** Demographics of Study Participants.

Demographics		
**Number of Participants**	18	
**Age (mean ± SD)**	12.2 ± 5.2	Range = 4.4 years–21.5 years
**Participant gender**	Male: 16 (88.9%)	Female: 2 (11.1%)
**Verbal or nonverbal**	Verbal: 16 (88.9%)	Nonverbal: 2 (11.1%)
**Social Communication Questionnaire (SCQ) Score (mean ± SD)**	18.8 ± 6.75	Range = 6–28

**Table 3:** Negative Effects. Issues reported by users or caregivers during the testing session are reported below.

Negative Effects	User (%, *n*)	Caregiver (%, *n*)	Notes
**Gastrointestinal (nausea, vomiting)**	0%, 0	0%, 0	None reported
**Ophthalmic (eye strain, dry eyes, changes in vision)**	6.3%, 1	0%, 0	Eye strain complaint, user took 20 s break and continued without further complaint
**Motor (trips, falls, abnormal motor movements)**	0%, 0	0%, 0	None reported
**Behavioral (tantrums, meltdowns)**	0%, 0	0%, 0	None reported
**Dermatologic (skin injury or burns, skin irritation)**	0%, 0	0%, 0	None reported
**Any complaint of discomfort**	6.3%, 1	0%, 0	Nose pieces initially caused one user discomfort.
**Minor neurological (headache, dizziness)**	6.3%, 1	0%, 0	One complaint of dizziness.
**Major neurological (seizures, dystonia, loss of consciousness)**	0%, 0	0%, 0	None reported

**Table 4:** Design concerns. Design concerns reported by users and by caregivers, including concerns raised spontaneously during or following testing session, as well as those mentioned in response to direct questions about design during structured interviews following testing sessions.

Design Concerns	User (%, *n*)	Caregiver (%, *n*)	Notes
**Smartglasses (hardware)**	25%, 4	12.5%, 2	Users and caregivers reported the smartglasses becoming warm after continued use
**Applications (software)**	0%, 0	0%, 0	None reported

## Abbreviations

AR	Augmented Reality
ASD	Autism Spectrum Disorder
BPAS	Brain Power Autism System
SCQ	Social Communication Questionnaire
VR	Virtual Reality
